# Genetic Evidence for Transboundary Circulation of Peste Des Petits Ruminants Across West Africa

**DOI:** 10.3389/fvets.2019.00275

**Published:** 2019-08-21

**Authors:** Kadidia Tounkara, Olivier Kwiatek, Mamadou Niang, Cheik Abou Kounta Sidibe, Amadou Sery, Martin Dakouo, Habib Salami, Modou Moustapha Lo, Aminata Ba, Mariame Diop, Ahmed Bezeid El Mamy, Ahmed Salem El Arbi, Yahya Barry, Ekaterina Isselmou, Habiboullah Habiboullah, Abdellahi Salem Lella, Baba Doumbia, Mohamed Baba Gueya, Joseph Savadogo, Lassina Ouattara, Germaine Minougou, Geneviève Libeau, Arnaud Bataille

**Affiliations:** ^1^CIRAD, UMR ASTRE, Montpellier, France; ^2^ASTRE, Univ. Montpellier, CIRAD, INRA, Montpellier, France; ^3^Laboratoire Central Vétérinaire, Bamako, Mali; ^4^Laboratoire National d'Elevage et de Recherches Vétérinaires (LNERV), Institut Sénégalais de Recherches Agricoles, Dakar-Hann, Sénégal; ^5^Office National de Recherches et de Développement de l'Elevage, Nouakchott, Mauritania; ^6^Ministère des Ressources Animales et Halieutiques, Ouagadougou, Burkina Faso

**Keywords:** virus spread, peste des petits ruminants, phylogeny, eradication, morbillivirus, small ruminant

## Abstract

Peste des Petits Ruminants (PPR) is a viral disease affecting predominantly small ruminants. Due to its transboundary nature, regional coordination of control strategies will be key to the success of the on-going PPR eradication campaign. Here, we aimed at exploring the extent of transboundary movement of PPR in West Africa using phylogenetic analyses based on partial viral gene sequences. We collected samples and obtained partial nucleoprotein gene sequence from PPR-infected small ruminants across countries within West Africa. This new sequence data was combined with publically available data from the region to perform phylogenetic analyses. A total of fifty-five sequences were obtained in a region still poorly sampled. Phylogenetic analyses showed that the majority of virus sequences obtained in this study were placed within genetic clusters regrouping samples from multiple West African countries. Some of these clusters contained samples from countries sharing borders. In other cases, clusters grouped samples from very distant countries. Our results suggest extensive and recurrent transboundary movements of PPR within West Africa, supporting the need for a regional coordinated strategy for PPR surveillance and control in the region. Simple phylogenetic analyses based on readily available data can provide information on PPR transboundary dynamics and, therefore, could contribute to improve control strategies. On-going and future projects dedicated to PPR should include extensive genetic characterization and phylogenetic analyses of circulating viral strains in their effort to support the campaign for global eradication of the disease.

## Introduction

Peste des petits ruminants (PPR) is a viral disease affecting predominantly small ruminants, such as sheep and goats. PPR is classified as a Transboundary Animal Disease (TAD) due to its rapid spread in large parts of Africa, the Middle East and Asia, associated with animal trade and human movements (1, 2). PPR is transmitted mostly through direct contact, spreading rapidly among immunologically naïve flocks with mortality rates reaching 90% (OIE Terrestrial Manual). Compulsory notification to the World Animal Health Organization (OIE) of the presence of PPR in a country leads to restriction on movements of livestock and animal products. Due to its impact on the livelihood and food security for smallholders farmers, the OIE and the Food and Agriculture Organization (FAO) have launched a campaign for the global eradication of PPR (3). For this transboundary disease, regional coordination of control strategies will be key to the success of the campaign. The re-emergence of PPR in Morocco, due to transboundary movement of infected animals, few years after complete eradication from the country stresses the importance of this aspect (4).

The extent of transboundary movements of PPR may be difficult to appreciate. Risks analyses based on animal mobility and animal trade data could guide the development of efficient national and regional surveillance and control strategies (5). In addition, viral genetic data from PPR-infected animals could provide direct evidence of the extent, location, and direction of the movements of the pathogen across borders, supporting data obtained from modeling analyses.

The virus causing PPR is a non-segmented, negative-sense RNA virus of the genus *Morbillivirus* in the family *Paramyxoviridae*. Recently, the International Committee on Taxonomy of Viruses (ICTV) has changed the name of the virus from *peste des petits ruminants virus* to *small ruminant morbillivirus* (6). Here, the abbreviation PPRV will be used throughout the text for the PPR virus to avoid confusing non-specialist readers interested in the global PPR eradication campaign. The PPRV genome has a length of 16 kb and encodes for 8 proteins: the nucleocapsid protein (N), the phosphoprotein (P), the matrix protein (M), the fusion protein (F), the haemagglutinin protein (H), the polymerase protein (L) and the two non-structural proteins, C and V (7). RNA viruses evolve rapidly, with a mutation rate between 10^−4^ and 10^−5^ mutations per base per replication cycle, accumulating genetic changes fast enough to be used to study epidemiological processes, notably thanks to the rise of high-throughput sequencing technologies (8). Based on genetic data, PPRV can be classified in 4 genetic lineages: lineages I and II in West Africa, lineage III in East Africa, and a lineage IV rapidly spreading in Asia, Middle East and in many parts of Africa (1, 9, 10). Interestingly, even simple phylogenetic analyses based on a short nucleotide segment of the N gene, obtained from a reverse transcription Polymerase Chain Reaction (RT-PCR) diagnostic method commonly-used for PPR detection and lineage identification (11), can provide some insights on transboundary movements of the disease. Notably, such analysis was used to follow the extension of lineage IV into West Africa (9). Similar analyses also provided some insights into the origin of the PPR emergence in Georgia (12). However, it has never been used to get insight into regional transboundary dynamics of PPR transmission in an endemic region.

Here, we aimed at exploring the extent of transboundary movement of PPR in West Africa using phylogenetic analyses based on partial N gene sequences. There are more than 160 million small ruminants in West Africa, with extensive livestock trade mostly from countries in the Sahel region toward southern West African countries (13, 14). PPR is endemic in West Africa, where it was first described in 1942 (15). Countries in the region implement vaccination against PPR but with limit success due to lack of funds and coordination (13). However, the implementation of regional projects such as the Regional Support Project Pastoralism in the Sahel (PRAPS, http://praps.cilss.int/) may improve the situation.

Due to transhumance and poorly controlled movement of animals across the region, we expect to find evidence of close phylogenetic relationship between PPRV strains in West Africa. We collected samples and obtained partial N gene sequences from PPRV-infected small ruminants across countries within West Africa. This new sequence data was combined with publically available data from the region to perform phylogenetic analyses and test this hypothesis.

## Materials and Methods

### Sampling and Laboratory Analysis

This study took advantage of sampling effort realized in the frame of previous projects or as part of routine control efforts carried out by veterinary services. Samples were collected from small ruminants showing clinical signs suggestive of PPR infection in the main markets of Dakar, Senegal in 2013, and in villages and markets in Burkina Faso, Ghana, Mali, and Mauritania in 2014 (Table 1). Veterinarians of national veterinary services conducted the field studies in accordance with local legislation, with no specific ethical approval required. Still, the tissues used in the study were sourced ethically. The study was conducted in animals in contact with outdoor environments with natural exposure to diseases (PPR is endemic in the region). Ocular or nasal swabs were collected on live animals by aseptic means and/or by non-invasive methods, and tissues (lung, lymph node and/or spleen) were sampled from animals that died of infection or were euthanized humanely if symptoms of acute PPR infection were observed (mucopurulent ocular/nasal discharges, diarrhea, fever, loss of weight, respiratory distress). The samples were kept at 4°C during the time of transport to the national veterinary laboratories. In addition, two positive samples collected in Mali in 1999 and in Burkina Faso in 2008, respectively, and stored at the FAO and OIE reference laboratory for PPR (CIRAD, Montpellier, France) were included in the study.

**Table 1:** List of samples used in this study.

**Location**	**Type**	**Species**	**Sample**	**N**	**Year**	**Accession number**
**Burkina faso**						
Binde	flock	sheep	Ns	2/5	2014	MK777897 MK777901
Ouindigui	flock	goat	Ns, Lg	0/3	2014	-
	flock	sheep	Ns	0/2	2014	-
Pibaore	flock	sheep	Ln	2/3	2014	MK777902
Sabou	flock	sheep	Ns	2/2	2014	failed
Toece	flock	sheep	Lg, Ln, Sp	0/3	2014	-
Zeguedeguin	flock	goat	Os	1/1	2008	MK777898
**Ghana**						
Atta Bagbe	flock	goat	Lg, Sp	2/3	2014	MK777904 MK777905
Ayensudo	flock	goat	Lg,Ln	1/2	2014	MK777904
Enyitsewdo	flock	goat	Lg, Ln, Sp	2/3	2014	MK777899
Wyamoah	flock	goat	Lg, Ln, Sp	4/4	2014	MK777905
Wyomoah	flock	goat	Lg, Ln, Sp	1/4	2014	MK777905
**Mali**						
Bamako	market	goat	Lg	1/1	1999	MK777896
Dialafara	market	goat	Os	1/1	2014	MK777888
Kolondieba	market	goat	Os	10/11	2014	MK777903 MK777894 MK777895
Tousseguela	market	goat	Os	3/3	2014	MK777892 MK777893
Samako	market	goat	Os	8/8	2014	MK777889 MK777890 MK777891
Sekou	market	goat	Os	5/5	2014	MK777887
**Mauritania**						
Atar	flock	goat	Os	1/8	2014	MK777900
	flock	sheep	Os	0/3	2014	-
**Senegal**						
Dakar	market	goat	Os	8/39	2013	MK777907
	market	sheep	Os	1/1	2013	MK777906
Total				57/115		21

*Location indicates the town where samples were collected or the main town closest to sampling site. Type indicates if samples were collected from flocks or in a market. N, number of positive samples/total samples tested. Lg, lung; Ln, lymph node; Os, ocular swab; Sp, spleen; failed, sequencing attempts failed; Accession number, GenBank accession number. Multiple accession numbers are given for one location when multiple non-identical sequences could be obtained from PPRV-positive samples. Accession numbers are repeated when the same sequence was obtained from several locations. Total of accession number is the number of non-identical sequences obtained*.

All samples collected were sent to CIRAD, Montpellier, France. Once there, the samples were processed in a biosafety level 3 containment laboratory.

At CIRAD, the tissue samples were cut to pieces and ground in 3 ml of Minimum Essential Media (MEM) by vortexing with 0.2 μm glass beads. The swabs were placed in 1 ml MEM and vortexed. In all cases, the sample suspensions were centrifuged 3 min at 1,000 g to collect the supernatant. Total RNA was extracted from the supernatant using the NucleoSpin RNA virus extraction Kit (Macherey-Nagel, France), according to the manufacturer's instructions.

A RT-PCR was performed using the qScript XLT One-Step RT-PCR Kit (Quantabio, VWR, France) to amplify a 351 base pair (bp) segment of the PPRV N gene with the NP3/NP4 primer pair modified from Couacy-Hymman et al. (11) (Forward NP3: 5′-GTC-TCG-GAA-ATC-GCC-TCA-CAG-ACT-3′ and Reverse NP4: 5′-CCT-CCT-CCT-GGT-CCT-CCA-GAA-TCT-3′) at a final concentration of 0.6 μM. PCR was set up under the following programme: 50°C for 30 min; 95°C for 15 min and 40 amplification cycles (10 s at 95°C, 30 s at 60°C and 30 s at 72°C) and a final extension step at 72°C for 5 min. The PCR products were resolved on 1.5% agarose gel to reveal the expected band size.

### Sequencing and Phylogenetic Analysis

The clean-up and sequencing of positive PCR products in both forward and reverse directions were carried out by Cogenics (France) or Genewiz (United Kingdom). The sequences were submitted to GenBank (Table 1). Forward and reverse DNA sequences were assembled using Geneious v. 8.1.6, and trimmed to remove poor-quality portions of the sequences (final size = 255 bp). Corrected sequences were aligned with 27 PPRV N gene sequences publicly available in GenBank using MEGA 6 (see Supplementary Material). This dataset contained representatives of the four genetic lineages, including 18 sequences of the lineage II, dominant in West Africa. A phylogenetic tree was constructed using the Neighbor-Joining and the Maximum Likelihood methods as implemented in MEGA 6, with node supports evaluated by bootstrap analyses (1,000 replicates).

## Results

A total of 115 samples from goat (*N* = 96) and sheep (*N* = 19) with suspicion of PPR infection were collected for this study (Table 1). Among the samples tested, 57 gave positive results by RT-PCR: 7 from Burkina Faso, 10 from Ghana, 28 from Mali, 1 from Mauritania, and 9 from Senegal. A partial N gene sequence was obtained from all samples except two samples from Sabou in Burkina Faso (Table 1). A total of 21 different partial N gene sequences were identified (Genbank accession numbers: MK777887-MK777907; Table 1).

**Figure 1:**
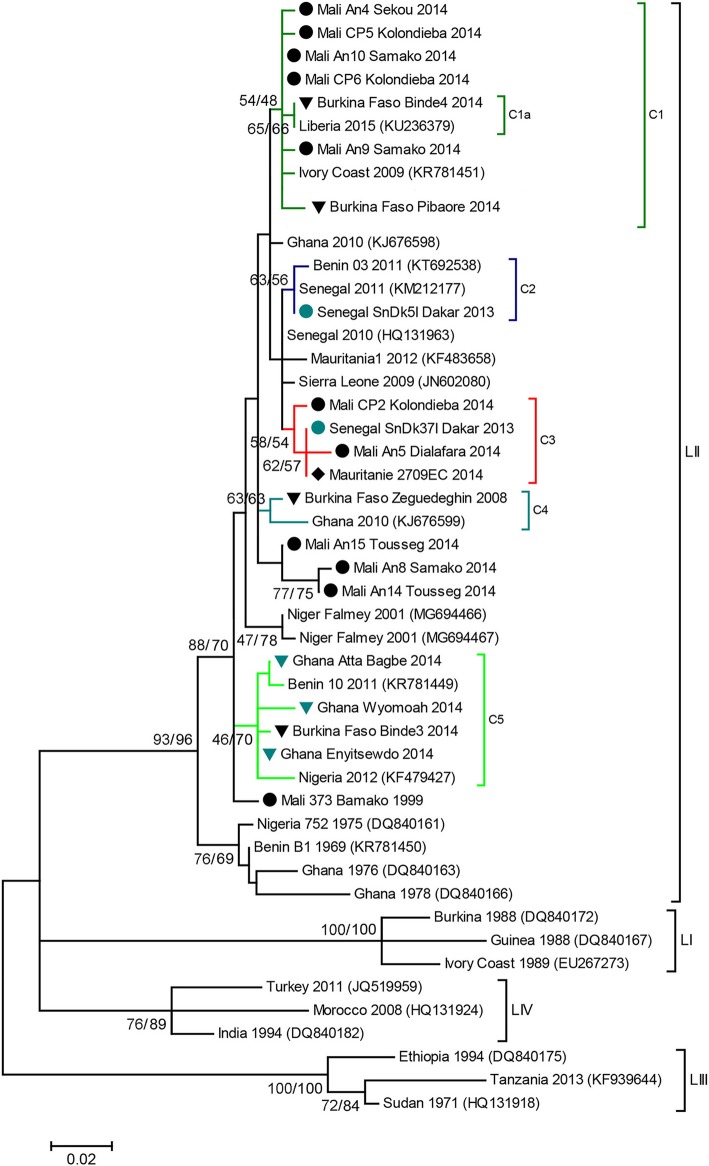
PPR N gene phylogenetic analysis. Phylogenetic tree constructed using a Maximum Likelihood inference method and showing the relationship based on N gene sequences of peste des petits ruminants virus (PPRV) samples, with a special focus on West Africa. Samples collected in this study are indicated by icons according to sampling location (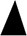 Burkina Faso, 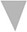
Ghana, 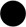
Mali, 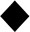
Mauritania, 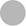
Senegal). Genetic clusters of interest to this study are indicated with colored branches, and named C1 to C5. The numbers at the nodes are bootstrap values obtained from 1,000 replicates (Neighboring-Joining/ Maximum Likelihood methods). Bootstrap values are shown if >50% for at least one inference method.

**Figure 2:**
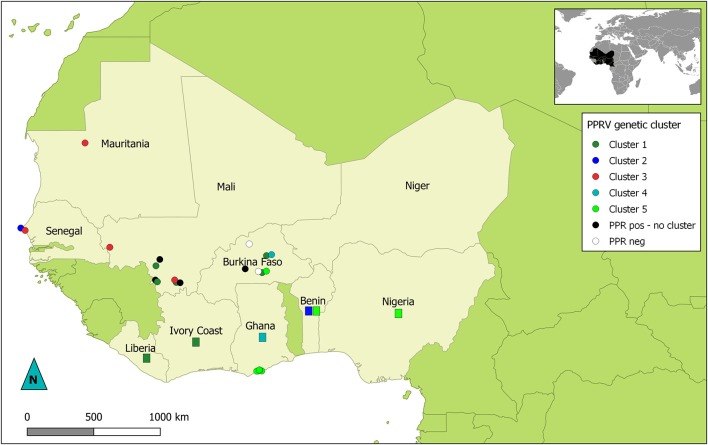
Map of West Africa showing sampling location according to their PPRV lineage II genetic cluster. Dots represent location of samples obtained for this study. Rectangles indicate countries of origin for publically available sequence data used in this study and belonging to genetic clusters of interest in this study. Dots and rectangles are colored according to the genetic cluster (C1 to C5) they were placed in by phylogenetic analysis (see Figure 1). Black dots represent PPRV positive samples with no specified genetic cluster. White dots indicate sampling sites where no PPRV positive samples were obtained.

Phylogenetic analyses showed that all the sequences obtained belonged to the lineage II (LII) of PPRV (Figure 1). The sample collected in Mali in 1999 was positioned at the base of all the LII samples collected in 2000–2014. Despite the short length of the sequences aligned (255 bp), multiple genetic clusters could be observed in the phylogenetic trees, with moderate (54–66%) or good (70–87%) bootstrap support for one or two inference method used (Figure 1). All sequences obtained in this study, except three samples from Mali, were placed within one of five genetic clusters regrouping samples from multiple West African and Central African countries (C1-C5 in Figures 1, 2). Cluster 1 included samples from Mali and Burkina Faso obtained in this study and samples from Liberia and Ivory Coast. Within this cluster, close phylogenetic relationship was observed between two samples from Burkina Faso and Liberia (Cluster 1a). One sample from Dakar belonged to Cluster 2 with other samples from Senegal and Benin. Samples from Mali, Senegal and Mauritania formed Cluster 3. The sample collected in Burkina Faso in 2008 clustered with a sample from Ghana (Cluster 4). Finally, sequences obtained in this study from Ghana and Burkina Faso formed the Cluster 5 with samples from Benin and Nigeria (Figures 1, 2). Bootstrap support was highest for Cluster 1b (87%) and 5 (70%).

## Discussion

A small region of the N gene (11) of PPRV is used to follow the changes in distribution of the four lineages across continents (1, 16, 17). Phylogenetic analyses based on this short (250–300 bp) sequence has been corroborated consistently by those defined on partial or full PPRV genome sequences in the recent years (18–20). A large amount of partial N gene sequence data are publically available because it is produced from one of the most common PPRV diagnostic method used worldwide (11) comparatively to the F gene (21). Therefore, this wealth of data could be used to further our understanding of the epidemiology and transmission dynamics of this important transboundary animal disease.

In this study, we focused on West Africa, aiming at exploring the extent of transboundary movement of PPRV in the region using phylogenetic analysis. PPRV sequences were obtained from fifty-fivesamples collected during this study, with a total of twenty-one new partial PPRV sequences identified in a region still poorly sampled. Most sequences were obtained from samples collected from goats, possibly because goats are usually more affected by PPR than sheep (7). All sequences obtained belonged to PPRV lineage II. Based on the limited number of samples available, it is hard to assess whether the Asiatic lineage IV, currently spreading into West Africa (9), was present in the countries sampled in 2013 and 2014. Further sampling, notably at the borders between Burkina Faso, Mali, Nigeria, Niger and Benin would be necessary to follow closely the progression of the lineage. In the same way, sampling size is too limited to evaluate if the lineage I, not reported since 2001 in Niger (9), was circulating in the regions sampled. Another potential bias is that sampling based on disease report may miss strains circulating silently without provoking any clear symptoms in the animals. Still, our results suggest that lineage II was the most dominant genetic lineage in the region at the time of sampling, as it has been observed before (1). The current distribution of PPRV genetic lineages in the region may be very different, notably because of the risk of rapid spread of the lineage IV in West Africa (9).

Phylogenetic analyses based on sequence data from this lineage can be used to study transboundary PPRV dynamics in the West African region, characterized by complex transboundary movements of animals through transhumance and trade. Indeed, our results showed that we could identify different genetic clusters within lineage II containing samples from more than one West African country, although good statistical support was obtained for only two of them. Some of these clusters consisted of samples from countries sharing borders (for example cluster 5 with samples from Ghana, Burkina Faso, Benin and Nigeria; Figure 2). In other cases, clusters grouped samples from very distant countries. This is the case for cluster 1a, which suggest virus circulation between Burkina Faso and Liberia (~1,500 km). Livestock trade between West African countries is extensive, with movement generally going from producers in the Sahel region toward southern West African countries (14). Our results corroborate previous studies highlighting the risk of intraregional trade for disease emergence (22).

The short sequences used in this study do not provide enough resolution to ascertain the relevance of some clusters identified or to perform complex phylogeographic and phylodynamic inferences that would inform us on the direction and intensity of the movement of PPRV. Some sequences included in the phylogenetic analyses could not be grouped within specific clusters, because of lack of resolution or the paucity of sequence data form the region. Still, our results clearly suggest extensive transboundary movements of PPRV within the region. The genetic clusters contain samples collected from 2004 to 2014 (Figure 1). It suggests that extensive movements are recurrent and not extraordinary events, although it is well-known that risk of virus spread may increase during specific religious events such as Tabaski (5). Our results support the call from the FAO and OIE for a regional coordinated strategy for the surveillance and control of the disease in order to eradicate it from West Africa (3). If vaccination campaigns are not coordinated in West Africa, it is likely that local efforts may be wasted due to the high risk of PPRV re-emergence through highly porous national borders, as happened in Morocco (4). Fortunately, the support for PPRV control efforts has increased, notably through regional projects such as the Regional Support Project Pastoralism in the Sahel (PRAPS, http://praps.cilss.int/) in West Africa. Such project should put emphasis into ensuring coordination among participating countries.

Risk mapping analyses based on national and regional animal trade and mobility data are important in the development and implementation of efficient surveillance strategy at national borders and at PPRV transmission hotspots. Our study shows that simple phylogenetic analyses based on readily available data can provide further information on PPRV transboundary dynamics and, therefore, could contribute to improve control strategies. On-going and future projects dedicated to PPRV should include extensive genetic characterization and phylogenetic analyses of circulating PPRV strains in their effort to support the campaign for global eradication of the disease.

## Data Availability

The datasets generated for this study can be found in Genbank, MK777887-MK777907.

## Ethics Statement

Veterinarians of national veterinary services conducted the field studies in accordance with local legislation, with no specific ethical approval required. Still, the tissues used in the study were sourced ethically. The study was conducted in animals in contact with outdoor environments with natural exposure to diseases (PPR is endemic in the region). Ocular or nasal swabs were collected on live animals by aseptic means and/or by non-invasive methods, and tissues (lung, lymph node and/or spleen) were sampled from animals that died of infection or were euthanized humanely if symptoms of acute PPR infection were observed (mucopurulent ocular/nasal discharges, diarrhea, fever, loss of weight, respiratory distress).

## Author Contributions

AmB, ABE, AS, ASE, AL, BD, CA, EI, GM, HH, HS, JS, LO, MG, MDi, ML, MN, and YB organized and carried out field examination of animals and sample collection. AA, KT, HS, MDa, and OK performed laboratory analyses. ArB, KT, OK, and HS performed phylogenetic analyses. ArB wrote the manuscript. ArB and GL designed the study.

### Conflict of Interest Statement

The authors declare that the research was conducted in the absence of any commercial or financial relationships that could be construed as a potential conflict of interest. The handling Editor declared a past co-authorship with one of the authors GL.
