# Corrigendum: Genetic Evidence for Transboundary Circulation of Peste Des Petits Ruminants Across West Africa

**DOI:** 10.3389/fvets.2019.00380

**Published:** 2019-10-30

**Authors:** Kadidia Tounkara, Olivier Kwiatek, Mamadou Niang, Cheik Abou Kounta Sidibe, Amadou Sery, Martin Dakouo, Habib Salami, Modou Moustapha Lo, Aminata Ba, Mariame Diop, Ahmed Bezeid El Mamy, Ahmed Salem El Arbi, Yahya Barry, Ekaterina Isselmou, Habiboullah Habiboullah, Abdellahi Salem Lella, Baba Doumbia, Mohamed Baba Gueya, Joseph Savadogo, Lassina Ouattara, Germaine Minougou, Geneviève Libeau, Arnaud Bataille

**Affiliations:** ^1^CIRAD, UMR ASTRE, Montpellier, France; ^2^ASTRE, Univ. Montpellier, CIRAD, INRA, Montpellier, France; ^3^Laboratoire Central Vétérinaire, Bamako, Mali; ^4^Laboratoire National d'Elevage et de Recherches Vétérinaires (LNERV), Institut Sénégalais de Recherches Agricoles, Dakar-Hann, Sénégal; ^5^Office National de Recherches et de Développement de l'Elevage, Nouakchott, Mauritania; ^6^Ministère des Ressources Animales et Halieutiques, Ouagadougou, Burkina Faso

**Keywords:** virus spread, peste des petits ruminants, phylogeny, eradication, morbillivirus, small ruminant

In the original article, there was an error. A sequence was mistakenly labeled as originating from the Central African Republic (GenBank accession number HQ131960) but was actually a duplicate of the sequence Burkina Faso Pibaore 2014, obtained during this study.

A correction has been made to the **Abstract**:

“Peste des Petits Ruminants (PPR) is a viral disease affecting predominantly small ruminants. Due to its transboundary nature, regional coordination of control strategies will be key to the success of the on-going PPR eradication campaign. Here, we aimed at exploring the extent of transboundary movement of PPR in West Africa using phylogenetic analyses based on partial viral gene sequences. We collected samples and obtained partial nucleoprotein gene sequence from PPR-infected small ruminants across countries within West Africa. This new sequence data was combined with publically available data from the region to perform phylogenetic analyses. A total of fifty-five sequences were obtained in a region still poorly sampled. Phylogenetic analyses showed that the majority of virus sequences obtained in this study were placed within genetic clusters regrouping samples from multiple West African countries. Some of these clusters contained samples from countries sharing borders. In other cases, clusters grouped samples from very distant countries. Our results suggest extensive and recurrent transboundary movements of PPR within West Africa, supporting the need for a regional coordinated strategy for PPR surveillance and control in the region. Simple phylogenetic analyses based on readily available data can provide information on PPR transboundary dynamics and, therefore, could contribute to improve control strategies. On-going and future projects dedicated to PPR should include extensive genetic characterization and phylogenetic analyses of circulating viral strains in their effort to support the campaign for global eradication of the disease.”

A correction has been made to the **Introduction**, paragraph five:

“Due to transhumance and poorly controlled movement of animals across the region, we expect to find evidence of close phylogenetic relationship between PPRV strains in West Africa. We collected samples and obtained partial N gene sequences from PPRV-infected small ruminants across countries within West Africa. This new sequence data was combined with publically available data from the region to perform phylogenetic analyses and test this hypothesis.”

A correction has been made to the **Results**, paragraph two:

“Phylogenetic analyses showed that all the sequences obtained belonged to the lineage II (LII) of PPRV (Figure 1). The sample collected in Mali in 1999 was positioned at the base of all the LII samples collected in 2000–2014. Despite the short length of the sequences aligned (255 bp), multiple genetic clusters could be observed in the phylogenetic trees, with moderate (54–66%) or good (70–87%) bootstrap support for one or two inference method used (Figure 1). All sequences obtained in this study, except three samples from Mali, were placed within one of five genetic clusters regrouping samples from multiple West African and Central African countries (C1-C5 in Figures 1, 2). Cluster 1 included samples from Mali and Burkina Faso obtained in this study and samples from Liberia and Ivory Coast. Within this cluster, close phylogenetic relationship was observed between two samples from Burkina Faso and Liberia (Cluster 1a). One sample from Dakar belonged to Cluster 2 with other samples from Senegal and Benin. Samples from Mali, Senegal and Mauritania formed Cluster 3. The sample collected in Burkina Faso in 2008 clustered with a sample from Ghana (Cluster 4). Finally, sequences obtained in this study from Ghana and Burkina Faso formed the Cluster 5 with samples from Benin and Nigeria (Figures 1, 2). Bootstrap support was highest for Cluster 1b (87%) and 5 (70%).”

**Figure 1:**
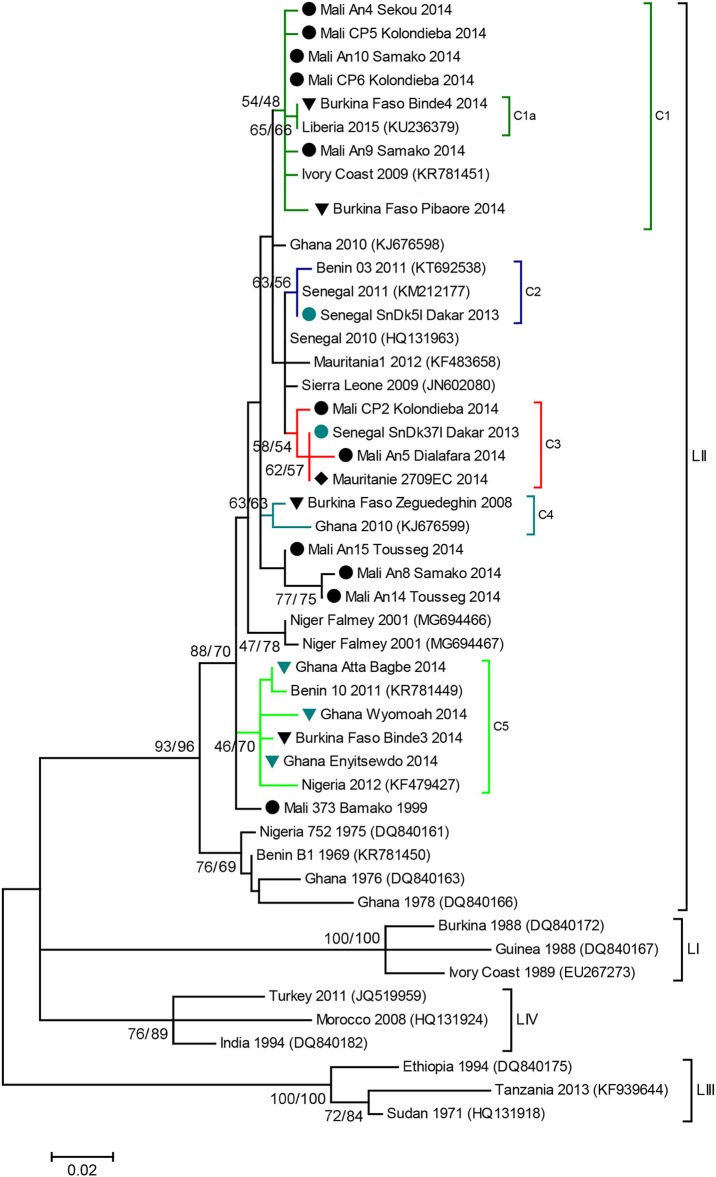
PPR N gene phylogenetic analysis. Phylogenetic tree constructed using a Maximum Likelihood inference method and showing the relationship based on N gene sequences of peste des petits ruminants virus (PPRV) samples, with a special focus on West Africa. Samples collected in this study are indicated by icons according to sampling location (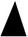
Burkina Faso, 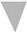
Ghana, 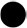
Mali, 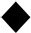
Mauritania, 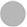
Senegal). Genetic clusters of interest to this study are indicated with colored branches, and named C1 to C5. The numbers at the nodes are bootstrap values obtained from 1,000 replicates (Neighboring-Joining/ Maximum Likelihood methods). Bootstrap values are shown if >50% for at least one inference method.

**Figure 2:**
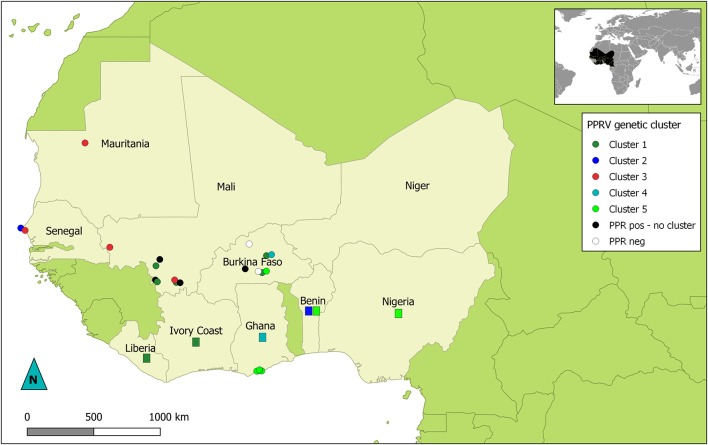
Map of West Africa showing sampling location according to their PPRV lineage II genetic cluster. Dots represent location of samples obtained for this study. Rectangles indicate countries of origin for publically available sequence data used in this study and belonging to genetic clusters of interest in this study. Dots and rectangles are colored according to the genetic cluster (C1 to C5) they were placed in by phylogenetic analysis (see Figure 1). Black dots represent PPRV positive samples with no specified genetic cluster. White dots indicate sampling sites where no PPRV positive samples were obtained.

A correction has been made to the **Discussion**, paragraph three:

“Phylogenetic analyses based on sequence data from this lineage can be used to study transboundary PPRV dynamics in the West African region, characterized by complex transboundary movements of animals through transhumance and trade. Indeed, our results showed that we could identify different genetic clusters within lineage II containing samples from more than one West African country, although good statistical support was obtained for only two of them. Some of these clusters consisted of samples from countries sharing borders (for example cluster 5 with samples from Ghana, Burkina Faso, Benin and Nigeria; Figure 2). In other cases, clusters grouped samples from very distant countries. This is the case for cluster 1a, which suggest virus circulation between Burkina Faso and Liberia (~1,500 km). Livestock trade between West African countries is extensive, with movement generally going from producers in the Sahel region toward southern West African countries (14). Our results corroborate previous studies highlighting the risk of intraregional trade for disease emergence (22).”

Lastly, corrections have been made to Figure 1 and Figure 2. The corrected figures appear below.

The authors apologize for these errors and state that this does not change the scientific conclusions of the article in any way. The original article has been updated.

