# Supplementary material for: Genetic Evidence for Transboundary Circulation of Peste Des Petits Ruminants Across West Africa
Source: Front Vet Sci. 2019 Aug 21;6:275. doi: 10.3389/fvets.2019.00275 (PMC6713047; doi:10.3389/fvets.2019.00275)
Supplement: Supplementary file 1 [file Data_Sheet_1.docx]

>Mali_Sekou_2014

CCTTCCTCCAGCACAAAATAGGTGAGGGAGAGTCGCCTACACCAGCAACCAAAGAAGAAG

TCAAAGCTGCGATCCCAAACGAATCTGAAGGAAGGGACATAAAGCGAACACGCCCAGGGA

AGCCCAGAGGAGAAACTCCCGGGCAACTGCTTCTGGAGATCATGCCAGAGGATGGAGTCT

CGCGAGAGTCTGGTCAAAACCCTCGTGAGGCTCAAAGATCGGCTGAGGCACTCTTCAGGC

TGCAGGCCATGGCCA

>Mali_Dialafara5_2014

CCTTCCTCCAGCACAAAATAGGTGAGGGAGAGTCGCCTACACCAGCGACCAAAGAAGAAG

TCAAAGCTGCGATCCCAAACGGATCTGAAGGAAGAGACACAAAACGAACACGCCCAGGGA

AGCCCAGAGGAGAAACTCCCGGGCAACTGCTTCTGGAGATCATGCCAGAGGATGGAGTCT

CGCGAGAGTCTGGTCAAAGCCCTCGTGAGGCTCAAAGATCGGCTGAGGCACTTTTCAGGC

TGCAGGCCATGGCCA

>Mali_Samako8_2014

CCTTCCTCCAGCACAAAACAGGCGAGGGAGAGTCGCCTACACCAGCGACCAAAGAAGAAG

TCAAAGCTGCGATCCCAAACGGATCCGAAGGAAGGGACATAAAGCGAACACGCCCAGGGA

AGCCCAGAGGAGAAACTCCCGGGCAACTGCTTCTGGAGATCATGCCAGAGGATGAATTCT

CGCGAGAGTCTGGTCAAAACCCTCGTGAGGCTCAAAGATCGGCTGAGGCACTCTTCAGGC

TGCAGGCCATGGCCA

>Mali_Samako9_2014

CCTTCCTCCAGCACAAAATAGGTGAGGGAGAGTCGCCTACACCAGCAACCAAAGAAGAAG

TCAAAGCTGCGATCCCAAACGGATCTGAAGGAAGGGACATAAAGCGAACACGCCCAGGGA

AGCCCAGAGGAGAAACTCCCGGGCAACTGCTCCTGGAGATCATGCCAGAGGATGGAGTCT

CGCGAGAGTCTGGTCAAAACCCTCGTGAGGCTCAAAGATCGGCTGAGGCACTCTTCAGGC

TGCAGGCCATGGCCA

>Mali_Samako10_2014

CCTTCCTCCAGCACAAAATAGGTGAGGGAGAGTCGCCTACACCAGCAACCAAAGAAGAAG

TCAAAGCTGCGATCCCAAACGGATCTGAAGGAAGGGACATAAAGCGAACACGCCCAGGGA

AGCCCAGAGGAGAAACTCCCGGGCAACTGCTTCTGGAGATCATGCCAGAGGATGGAGTCT

CGCGAGAGTCTGGTCAAAACCCTCGTGAGGCTCAAAGATCGGCTGAGGCACTCTTCAGGC

TGCAGGCCATGGCCA

>Mali_Tousseguela14_2014

CCTTCCTCCAGCACAAAACAGGCGAGGGAGAGTCGCCTACACCAGCGACCAAAGAAGGAG

TCAAAGCTGCGATCCCAAACGGATCCGAAGGAAGGGACATAAAGCGAACACGCCCAGGGA

AGCCCAGAGGAGAAACTCCCGGGCAACTGCTTCTGGAGATCATGCCAGAGGATGAATTCT

CGCGAGAGTCTGGTCAAAACCCTCGTGAGGCTCAAAGATCGGCTGAGGCACTCTTCAGGC

TGCAGGCCATGGCCA

>Mali_Tousseguela15_2014

CCTTCCTCCAGCACAAAATAGGTGAGGGAGAGTCGCCTACACCAGCGACCAAAGAAGGAG

TCAAAGCTGCGATCCCAAACGGATCTGAAGGAAGGGACATAAAGCGAACACGCCCAGGGA

AGCCCAGAGGAGAAACTCCCGGGCAACTGCTTCTGGAGATCATGCCAGAGGATGAATTCT

CGCGAGAGTCTGGTCAAAACCCTCGTGAGGCTCAAAGATCGGCTGAGGCACTCTTCAGGC

TGCAGGCCATGGCCA

>Mali_Kolondieba5_2014

CCTTCCTCCAGCACAAAATAGGTGAGGGAGAGTCGCCTACACCAGCAACCAAAGAAGAAG

TCAGAGCTGCGATCCCAAACGGATCTGAAGGAAGGGACATAAAGCGAACACGCCCAGGGA

AGCCCAGAGGAGAAACTCCCGGGCAACTGCTTCTGGAGATCATGCCAGAGGATGGAGTCT

CGCGAGAGTCTGGTCAAAACCCTCGTGAGGCTCAAAGATCGGCTGAGGCACTCTTCAGGC

TGCAGGCCATGGCCA

>Mali_Kolondieba6_2014

CCTTCCTCCAGCACAAAATAGGTGAGGGAGAGTCGCCTACACCAGCAACCAAAGAAGAAG

TCAAAGCTGCGATCCCAAACGGATCTGAAGGAAGGGACATAAAGCGAACACGCCCAGGGA

AGCCCAGAGGAGAAACTCCCGGGCAACTGCTTCTGGAGATCATGCCAGAGGATGGAGTCT

CGCGAGAGTCTGGTCAAAACCCTCGTGAGGCTCAAAGATCGGCTGAGGCACTCTTCAGGC

TGCAGGCCATGGCCA

>Mali_Bamako373_1999

CCTTCCTCCAGCGCAAAATAGGTGAGGGAGAGTCGCCTACACCAGCGACCAAAGAAGAAG

TCAAAGCTGCGATCCCAAACGGGTCTGAAGGAAGGGACATGAAGCGAACACGCCCAGGGA

AGCCCAGAGGAGAAACTCCCGGCCAACTGCTTCTGGAGATCATGCCAGAGGATGAAGTCT

CGCGAGAGTCTGGTCAAAACCCTCGTGAGGCTCAAAGATCGGCTGAGGCACTCTTCAGGC

TGCAGGCCATGGCCA

>Burkina_Faso_Binde_2014

CCTTCCTCCAGCACAAAATAGGTGAGGGAGAGTCGCCTACACCAGCGACCAAAGAAGAAG

TCAAAGCTGCGATCCCAAACGGGTCCGAAGGAAGGGACATAAAGCGAACACGCCCAGGGA

AGCCCAGAGGAGAAACTCCCGGCCAACTGCTTCTGGATATCATGCCAGAGGATGAAGTCT

CGCGAGAGTCTGGTCAAAACCCTCGTGAGGCTCAAAGATCGGCTGAGGCACTTTTCAGGC

TGCAGGCCATGGCCA

>Sierra_Leone_2009_(JN602080)

CCUUCCUCCAGCACAAAAUAGGUGAGGGAGAGUCGCCUACACCAGCGACCAAAGAAGAAG

UCAAAGCUGUGAUCCCAAACGGAUCUGAAGGAAGAGACAUAAAGCGAACACGCCCAGGGA

AGCCCAGAGGAGAAACUCCCGGGCAACUGCUUCUGGAGAUCAUGCCAGAGGAUGGAGUCU

CGCGAGAGUCUGGUCAAAACCCUCGUGAGGCUCAAAGAUCGGCUGAGGCACUCUUCAGGC

UGCAGGCCAUGGCCA

>Burkina_Faso_Zegued_2008

CCTTCCTCCAGCACAAAATAGGTGAGGGAGAGTCGCCTACACCAGCGACCAAAGAAGAAG

TTAAAGCTGCGATCCCAAACGGATCTGAAGGAAGGGACATAAAGCGAACACGCCCAGGGA

AGCCCAGAGGAGAAACTCCCGGGCAACTGCTTCTGGAGATCATGCCAGAGGATGAAGTCT

CGCGAGAGTCTGGTCAAAACTCTCGTGAGGCTCAAAGATCGGCTGAGGCACTCTTCAGGC

TGCAGGCCATGGCCA

>Ghana_Enyitsewdo_2014

CCTTCCTCCAGCACAAAATAGGTGAGGGAGAGTCGCCTACACCAGCGACCAAAGAAGAAG

TCAAAGCTGCGATCCCAAACGGGTCCGAAGGAAGGGACATAAAGCGAACACGCCCAGGGA

AGCCCAGAGGAGAAACTCCCGGCCAACTGCTTCTGGAGATCATGCCAGAGGATGAAGTCT

CGCGAGAGTCTGGTCAAAACCCTCGTGAGGCTCAAAGATCGGCTGAGGCACTTTTCAGGC

TGCAGGCCATGGCCA

>Mauritanie_Atar_2014

CCTTCCTCCAGCACAAAATAGGTGAGGGAGAGTCGCCTACACCAGCGACCAAAGAAGAAG

TCAAAGCTGCGATCCCAAACGGATCTGAAGGAAGAGACACAAAACGAACACGCCCAGGGA

AGCCCAGAGGAGAAACTCCCGGGCAACTGCTTCTGGAGATCATGCCAGAGGATGGAGTCT

CGCGAGAGTCTGGTCAAAACCCTCGTGAGGCTCAAAGATCGGCTGAGGCACTCTTCAGGC

TGCAGGCCATGGCCA

>Central_African_Replublic_2004_(HQ131960)

CCTTCCTCCAGCACAAAATAGGTGAGGGAGAGTCGCCTACACCAGCAACCAAAGAAGAAG

TCAAAGCTGCGATCCCAAACGGATCTGAAGGAAGGGACACAAAGCGAACACGCCCAGGGA

AGCCCAGAGGAGAAACTCCCGGGCAACTGCTTCTGGAGATCATGCCAGAGGATGGAGTCT

CGCGAGAGTCGGGTCAAAACCCTCGTGAGGCTCAAAGATCGGCTGAGGCACTCTTCAGGC

TGCAGGCCATGGCCA

>Niger_2001_(MG694466)

CCTTCCTCCAGCACAAAACAGGTGAGGGAGAGTCGCCTACACCAGCGACCAAAGAAGAAG

TCAAAGCTGCGATCCCAAACGGATCCGAAGGAAGGGACATAAAGCGAACACGCCCAGGGA

AGCCCAGAGGAGAAACTCCCGGCCAACTGCTTCTGGAGATCATGCCAGAGGATGAAGTCT

CGCGAGAGTCTGGTCAAAACCCTCGTGAGGCTCAAAGATCGGCTGAGGCACTCCTCAGGC

TGCAGGCCATGGCCA

>Niger_2001_(MG694467)

CCTTCCTCCAGCACAAAACAGGTGAGGGAGAGTCGCCTACACCAGCGACCAAAGAAGAAG

TCAAAGCTGCGATCCCAAACGGATCCGAAGGAAGGGACATAAAGCGAACACGCCCAGGGA

AGCCCAGAGGAGAAACTCCCGGCCAACTGCTTCTGGAGATCATGCCAGAGGATGAACTCT

CGCGAGAGTCTGGTCAAAACCCTCGTGAGGCTCAAAGATCGGCTGAGGCACTCCTCAGGC

TGCAGGCCATGGCCA

>Burkina_Faso_Binde4_2014

CCTTCCTCCAGCACAAAATAGGTGAGGGAGAGTCGCCTACACCAGCAACCAAAGAAGAAG

TCAAAGCTGCGATCCCAAACGGATCTGAAGGAAGGGACATAAAGCGAACACGCCCAGGGA

AGCCCAGAGGAGAAACTCCCGGGCAACTGCTTCTGGAGATCATGCCAGAGGATGGAGTCT

CGCGAGAGTCTGGTCAGAACCCTCGTGAGGCTCAAAGATCGGCTGAGGCACTCTTCAGGC

TGCAGGCCATGGCCA

>Burkina_Faso_Pibaore_2014

CCTTCCTCCAGCACAAAATAGGTGAGGGAGAGTCGCCTACACCAGCAACCAAAGAAGAAG

TCAAAGCTGCGATCCCAAACGGATCTGAAGGAAGGGACACAAAGCGAACACGCCCAGGGA

AGCCCAGAGGAGAAACTCCCGGGCAACTGCTTCTGGAGATCATGCCAGAGGATGGAGTCT

CGCGAGAGTCGGGTCAAAACCCTCGTGAGGCTCAAAGATCGGCTGAGGCACTCTTCAGGC

TGCAGGCCATGGCCA

>Mali_Kolondieba2_2014

CCTTCCTCCAGCACAAAATAGGTGAGGGAGAGTCGCCTACACCAGCGACCAAAGAAGAAT

TCAAAGCTGCGATCCCAAACGGATCTGAAGGAAGAGACATAAAACGAACACGCCCAGGGA

AGCCCAGAGGAGAAACTCCCGGGCAACTGCTTCTGGAGATCATGCCAGAGGATGGAGTCT

CGCGAGAGTCTGGTCAAAACCCTCGTGAGGCTCAAAGATCGGCTGAGGCACTCTTCAGGC

TGCAGGCCATGGCCA

>Ghana_Atta_Bagbe_2014

CCTTCCTCCAGCACAAAATAGGTGAGGGAGAGTCGCCTACACCAGCGACCAAAGAAGAAG

TCAAAGCTGCGATCCCAAACGGGTCCGAAGGAAGGGACATAAAGCGAACACGCCCAGGGA

AGCCCAGAGGAGAAACTCCCGGCCAACTGCTTCTGGAGATCATGCCAGAGGATGAAGTCT

CGCGAGAGTCTGGTCAAAACCCTCGTGAGGCTCAAAGATCGGCTGAGGCACTTTTTAGGC

TGCAGGCCATGGCCA

>Ghana_Wyomoah_2014

CCTTCCTCCAGCACAAAATAGGTGAGGGAGAGTCGCCTACACCAGCGACCAAAGAAGAAG

TCAAAGCTGCGATCCCAAACGGGTCCGAAGGAAGGGACATAAAGCGAACACGCCCAGGGA

AGCCCAGAGGAGAAACTCCCGGCCAACTGCTTCTGGAGATCATACCAGAGGATGAAGTCT

CGAGAGAGTCTGGTCAAAACCCTCGTGAGGCTCAAAGATCGGCTGAAGCACTTTTCAGGC

TGCAGGCCATGGCCA

>Ghana_1976_(DQ840163)

CCTTCCTCCAGCATAAAATAGATGAGGGAGAGTCGCCTACACCAGCGACCAGAGAAGAAT

TCAAAGCTACGATCCCAAATGGGTCTGAAGGAAGGGACACAAAGCGAACACGCTCAGGAA

AACCCAGAGGAGAAACTCCCGGCCAACTGCTTCTGGAGATCATGCCAGAGGATGAAGTCT

CGCGAGAGTCTAGTCAAAACCCTCGTGAGGCTCAAAGATCGGCTGAGGCACTCTTCAGGC

TGCAGGCCATGGCCA

>Ghana_1978_(DQ840166)

CCTTCCTCCAGCACAAAATAGATGAGGGAGATGCGCCTACACCAGCGACCAGAGAAGAAG

TCAAGGCTGCGATCCCAAATGGGTCCGAAGGAAGGGAGCCAAAGCGAACACGCTCAGGAA

AGCCCAGAGGAGAAACTCCCGGCCAACTGCTTCTGGAGATCATGCCAGAGGATGAAGTCT

CGCGAGAGTCTAGTCAAAACCCTCGTGAGGCTCAAAGATCGGCTGAGGCACTCTTCAGGC

TGCAGGCCATGGCCA

>Nigeria_752_1975_(DQ840161)

CCTTCCTCCAGCATAAAATAGATGAGGGAGAGTCGCCTACACCAGCGACCAGAGAAGAAG

TCAAAGCTGCGATCCCAAATGGGTCTGAAGGAAGGGACATAAAGCGAACACGCTCAGGGA

AGCCCAGAGGAGAAACCCCCGGCCAACTGCTTCTGGAGATCATGCCAGAGGATGAAGTCT

CGCGAGAGTCTAGTCAAAACCCTCGTGAGGCTCAAAGATCGGCTGAGGCACTCTTCAGGC

TGCAGGCCATGGCCA

>Benin_2011_(KR781449)

CCTTCCTCCAGCACAAAATGGGTGAGGGAGAGTCGCCTACACCAGCGACCAAAGAAGAAG

TCAAAGCTGCGATCCCAAACGGGTCCGAAGGAAGGGACATAAAGCGAACACGCCCAGGGA

AGCCCAGAGGAGAAACTCCCGGCCAACTGCTTCTGGAGATCATGCCAGAGGATGAAGTCT

CGCGAGAGTCTGGTCAAAACCCTCGTGAGGCTCAAAGAT---------------------

---------------

>Benin_B1_1969_(KR781450)

CCTTCCTCCAGCATAAAATAGATGAGGGAGAGTCGCCTACACCAGCGACCAGAGAAGAAG

TCAAAGCTGCGATCCCAAATGGGTCTGAAGGAAGGGACATAAAGCGAACACGCTCAGGAA

AGCCCAGAGGAGAAACTCCCGGCCAACTGCTTCTGGAGATCATGCCAGAGGATGAAGTCT

CGCGAGAGTCTAGTCAAAACCCTCGTGAGGCTCAAAGAT---------------------

---------------

>Benin_2011_(KT692538)

CCTTCCTCCAGCACAAAATAGGTGAGGGAGAGTCGCCTACACCAGCGACCAAAGAAGAAG

TCAAAGCTGCGATCCCAAACGGATCCGAAGGAAGAGACATAAAGCAAACACGCCCAGGGA

AGCCCAGAGGAGAAACTCCCGGGCAACTGCTTCTGGAGATCATGCCAGAGGATGGAGTCT

CGCGAGAGTCTGGTCAAAACCCTCGTGAGGCTCAAAGAT---------------------

---------------

>Ivory Coast_2009_(KR781451)

CCTTCCTCCAGCACAAAATAGGTGAGGGAGAGTCGCCTACACCAGCAACCAAAGAAGAAG

TCAAAGCTGCGATCCCAAACGGATCTGAAGGAAGGGACATAAAGCGAACACGCCCAGGGA

AGCCCAGAGGAGAAACTCCCGGGCAACTGCTTCTGGAGATCATGCCAGAGGATGGAGTCT

CGCGAGAGTCTGGTCAAAACCCTCGTGAGGCTCAAAGATCGGCTGAGGCACTTTTCAGGC

TGCAGGCCATGGCCA

>Senegal_2010_(HQ131963)

CCTTCCTCCAGCACAAAATAGGTGAGGGAGAGTCGCCTACACCAGCGACCAAAGAAGAAG

TCAAAGCTGCGATCCCAAACGGATCTGAAGGAAGAGACATAAAGCGAACACGCCCAGGGA

AGCCCAGAGGAGAAACTCCCGGGCAACTGCTTCTGGAGATCATGCCAGAGGATGGAGTCT

CGCGAGAGTCTGGTCAAAACCCTCGTGAGGCTCAAAGATCGGCTGAGGCACTCTTCAGGC

TGCAGGCCATGGCCA

>Nigeria_2012_(KF479427)

CCTTTCTCCAGCACAAAATAGGTGAGGGAGAGTCGCCTACACCAGCGACCAAAGAAGAAG

TCAAAGCTGCGATTCCGAACGGGTCCGAAGGAAGGGACATAAAGCGAACACGCCCAGGGA

AGCCCAGAGGAGAAACTCCCGGCCAACTGCTTCTGGAGATCATGCCAGAGGATGAAGTCT

CGCGAGAGTCTGGTCAAAACCCTCGTGAGGCTCAAAGATCGGCTGAGGCACTTTTCAGGC

TGCAGGCCATGGCCA

>Ghana_2010_(KJ676598)

CCTTCCTCCAGCACAAAATAGGTGAGGGAGAGTCGCCTACACCAGCGACCAAAGAAGAAG

TCAAAGCTGCAATCCCAAACGGATCTGAAGGAAGGGACATAAAGCGAACACGCCCAGGGA

AGCCCAGAGGAGAAACTCCCGGGCAACTGCTTCTGGAGATCATGCCAGAGGATGGAGTCT

CGCGAGAGTCTGGTCAAAACCCTCGTGAGGCTCAAAGATCGGCTGAGGCACTCTTCAGGC

TGCAGGCCATGGCCA

>Ghana_2010_(KJ676599)

CCTTCCTCCAGCACAAAATTGGTGAGGGAGAGTCGCCTACACCAGCGACCAAAGAAGAAG

TTAAAGCTGCGATCCCAAACGGATCTGAAGGAAGGGACATAAAGCGAACACGCCCAAGGA

AGCCCAGAGGAGAAACTCCCGGGCAACTGCTTCTGGAGATCATGCCAGAGGATGAAGCCT

CGCGAGAGTCTGGTCAAAACCCTCGTGAGGCTCAAAGATCGGCTGAGGCACTCTTCAGGC

TGCAGGCCATGGCCA

>Senegal_2011_(KM212177)

CCTTCCTCCAGCACAAAATAGGTGAGGGAGAGTCGCCTACACCAGCGACCAAAGAAGAAG

TCAAAGCTGCGATCCCAAACGGATCCGAAGGAAGAGACATAAAGCGAACACGCCCAGGGA

AGCCCAGAGGAGAAACTCCCGGGCAACTGCTTCTGGAGATCATGCCAGAGGATGGAGTCT

CGCGAGAGTCTGGTCAAAACCCTCGTGAGGCTCAAAGATCGGCTGAGGCACTCTTCAGGC

TGCAGGCCATGGCCA

>Burkina_Faso_1988_(DQ840172)

CTTTCCTCCAGCATAAAACAGATGAGGGAGAGTCATCTGCACCAGTGACCAGAGAAGAAG

TCAAGGCTGCGATTCCAAATGGGTCCGAAGAAAGGGACAAAAGGCGAACCCGCCCAGGAA

GGCCCAGAGGAGAAACCCCGAGCCAACCGCTCCTGGAAATCATGCCAGAGGATGAGGCCT

CGAGAGAATCCGGCCAAACCTCTCGTGAGGCTCAGAGGTCGGCCGAGGCACTCTTCAGGC

TGCAAGCTATGGCCA

>Ivory_Coast_1989_(EU267273)

CTTTCCTCCAGCACAAAACAGGAGAGGGAGAGTCATCTGCACCAGTGACCAGAGAAGAAG

TCAAGGCTGCGATTCCGAATGGGTCCGAAGAAAGGGACAAAAGGCGAACCCGCCCAGGAA

GGTCCAGAGGAGAAATCCCGAGCCAACCGCTCCTGGAAATCATGCCAGAGGATGAGGCCT

CGAGAGAATCCGGCCAAACCCCTCGTGAGGCTCAGAGGTCGGCTGAGGCACTCTTCAGGC

TGCAAGCTATGGCCA

>Guinea_1988_(DQ840167)

CTTTCCTCCAGCATAAAACGGGAGAGGGAGAGTCATCTGCACCAGTGACCAGAGAAGAAG

TCAAGACTGCGATTCCAAATGGGTCCGAAGAAAGGGACAAAAGGCGAGCCCGCTCAGGAA

GGCCCAGAGGAGAAACCCCGAGCCAACCGCTCCTGGAAATCATGCCAGAGGATGAGGCCT

CGGGGGAATCCGGTCAAACCCCTCGTGAGGCTCAGAGGTCGGCAGAGGCACTCTTCAGGC

TGCAAGCTATGGCCA

>Morocco_2008_(HQ131924)

CCTTCCTCCAGCACAAAACAGGAGAGGGAGATTCGCCCACACCAGCGACCAGAGAAGGGG

TCAAAGCTGCGATCTCAAACGGACCTGAAGAAAGGGATAGAAAGCAAACACGCCCAGGAA

GGCCCAGAGGAGAGACCCCCGGTCAACTGCTCCTGGAAATCATGCCAGAGGATGAGGTTC

CGCGAGAGTCTGGTCAAAACCCTCGTGAGGCTCAAAGATCAGCCGAGGCACTCTTCAGGC

TGCAGGCCATGGCCA

>Turkey_2011_(JQ519959)

CCTTCCTCCAGCACAAAACAGGAGAGGGAGAGTCGTCCGCACCAGCAACCAGAGAAGGGG

TCAAAGCTGCGATCCCAAACGGATCCGAAGAACGGGACAGAAAGCAAACACGCCCAGGAA

GGTCCAGAGGAGAGACCCCCAGCCAACTGCTCCTGGAAATCATGCCAGAAGATGAGGTCT

CGCGAGAGTCTGGTCAAAACCCTCGTGAGGCTCAAAGATCGGCTGAGGCACTCTTCAGGC

TGCAGGCCATGGCCA

>India_1994_(DQ840182)

CCTTCCTCCAGCACAAAACAGGAGAGGGAGAGTCGTCCGCACCAGCGACCAGAGAAGGGG

TCAAGGCTGCGATCCCAAACGGATCTGAAGAGAGGGACAGAAAGCAAACACGCCCAGGAA

GGCCCAGAGGAGAGACCCCCGGCCAACTGCTCCTGGAAATCATGCCAGAGGATGAGGTCT

CGCGAGAGTCTGGCCAAAACCCTCGTGAGGCTCAAAGATCGGCCGAGGCACTCTTCAGGC

TGCAGGCCATGGCCA

>Tanzania_2013_(KF939644)

CCTTCCTCCAGCATAAAATAGGAGAGGGAGAGTCACATGCATCGGCGACCAGGGAAGAAG

TCAAAGCTGCGACCCCAAATGGGCCCGACGAAAAGGACAAAACTCGGGGGCGCTCAGGAA

AGCCAAGAGGAGGAACCCCCGACCAACTGCTCCTAGAAATTATGCCTGAAGACGAAGTCC

CGCGAGGGTCTGGACAAAACCCTCGTGAGGCTCAAAGATCGGCCGAGGCACTCTTTAGAC

TGCAGGCCATGGCCA

>Sudan_1971_(HQ131918)

CCUUCCUCCAGCAUAAAAUAGGAGAGGGAGAGUCACAUGCAUCGGCGACCAGGGAAGAAG

UCAAAGCUGCGACCCCAAAUGGGCCCGACGAAAAGGACAAAAAACGAGCACGCUCAGGAA

GGCCAAGAGGAGGAACCCCCGACCAACUGCUCCUGGAAAUCAUGCCUGAAGACGAGGUCC

CGCGAGGGUCUGGACAAAACCCUCGUGAGGCUCAACGAUCGGCCGAGGCACUCUUUAGAC

UGCAGGCCAUGGCCA

>Ethiopia_1994_(DQ840175)

CCTTCCTCCAGCATAAAATGGGAGAAGGAGAGTCACATGCATCGGCGACCAGGGAAGAAG

CCAAAGCTGCGATCCCATATGGGCCCGACGAAAAGGACAAAAAACGAGCACGCTCAGGAA

GGCCAAGAGGAGGAACCCCCGACCAACTGCTTCTGGAAATCATGCCTGAAGATGAGGTCC

CGCGAGGGTCTGGACAAAACCCTCGTGAGGCTCAAAGATCGGCCGAGGCACTCTTCAGAC

TGCAGGCCATGGCCA

>Senegal_Dakar37_2013

CCTTCCTCCAGCACAAAATAGGTGAGGGAGAGTCGCCTACACCAGCGACCAAAGAAGAAG

TCAAAGCTGCGATCCCAAACGGATCTGAAGGAAGAGACACAAAACGAACACGCCCAGGGA

AGCCCAGAGGAGAAACTCCCGGGCAACTGCTTCTGGAGATCATGCCAGAGGATGGAGTCT

CGCGAGAGTCTGGTCAAAACCCTCGTGAGGCTCAAAGATCGGCTGAGGCACTCTTCAGGC

TGCAGGCCATGGCCA

>Senegal_Dakar5_2013

CCTTCCTCCAGCACAAAATAGGTGAGGGAGAGTCGCCTACACCAGCGACCAAAGAAGAAG

TCAAAGCTGCGATCCCAAACGGATCCGAAGGAAGAGACATAAAGCGAACACGCCCAGGGA

AGCCCAGAGGAGAAACTCCCGGGCAACTGCTTCTGGAGATCATGCCAGAGGATGGAGTCT

CGCGAGAGTCTGGTCAAAACCCTCGTGAGGCTCAAAGATCGGCTGAGGCACTCTTCAGGC

TGCAGGCCATGGCCA

>Mauritania1_2012_(KF483658)

CCTTCCTCCAGCACAAAATAGGTGAGGGAGAGTCGCCTACACCAGCGACCAAAGAAGAAG

TCAAAGCTGCGATCCCAAACGGATCTGAAGGAAGAGACATAAAGCGAACACGCCCAGGGA

AGCCCAGAGGAGAAACTCCCGGGCAACTGCTTCTGGAGATCATGCCAGAGGATGGAGTTT

CGCGAGAGTCTGGTCAAAACTCTCGTGAGGCTCAAAGATCGGCTGAGGCACTCTTCAGGC

TGCAGGCCATGGCCA

>Liberia_2015_(KU236379)

CCTTCCTCCAGCACAAAATAGGTGAGGGAGAGTCGCCTACACCAGCAACCAAAGAAGAAG

TCAAAGCTGCGATCCCAAACGGATCTGAAGGAAGGGACATAAAGCGAACACGCCCAGGGA

AGCCCAGAGGAGAAACTCCCGGGCAACTGCTTCTGGAGATCATGCCAGAGGATGGAGTCT

CGCGAGAGTCTGGTCAGAACCCTCGTGAGGCTCAAAGATCGGCTGAGGCACTCTTCAGGC

TGCAGGCCATGGCCA
